# PLIN5-mediated lipid droplet–mitochondria contacts dysregulation underlies the increased susceptibility to acute kidney injury with aging

**DOI:** 10.1093/lifemedi/lnag025

**Published:** 2026-07-08

**Authors:** Siyang Wang, Jie Gao, Zhuo Li, Yue Xu, Guangyan Cai, Xiangmei Chen

**Affiliations:** Department of Nephrology, the First Medical Center, Chinese PLA General Hospital, Chinese PLA Institute of Nephrology, State Key Laboratory of Kidney Diseases, National Clinical Research Center for Kidney Diseases, Beijing 100853, China; Department of Emergency Medicine, Xinqiao Hospital, Army Medical University (Third Military Medical University), Chongqing 400037, China; Department of Nephrology, Shandong Provincial Hospital Affiliated to Shandong First Medical University, Jinan 250021, China; Department of Nephrology, Shandong Provincial Hospital Affiliated to Shandong First Medical University, Jinan 250021, China; Department of Nephrology, the First Medical Center, Chinese PLA General Hospital, Chinese PLA Institute of Nephrology, State Key Laboratory of Kidney Diseases, National Clinical Research Center for Kidney Diseases, Beijing 100853, China; Department of Nephrology, the First Medical Center, Chinese PLA General Hospital, Chinese PLA Institute of Nephrology, State Key Laboratory of Kidney Diseases, National Clinical Research Center for Kidney Diseases, Beijing 100853, China; Department of Nephrology, the First Medical Center, Chinese PLA General Hospital, Chinese PLA Institute of Nephrology, State Key Laboratory of Kidney Diseases, National Clinical Research Center for Kidney Diseases, Beijing 100853, China

**Dear Editor,** 

Aging is a risk factor for acute kidney injury (AKI). Perilipin 5 (PLIN5), a key regulator bridging lipid droplets (LDs) and mitochondria, has not been previously investigated in renal aging. Our findings provide the first experimental evidence that PLIN5 downregulation-induced impairment of LD–mitochondria contacts may play a critical role in age-related AKI susceptibility.

The incidence of AKI has increased annually. Age is a significant risk factor for AKI. Although mechanistic studies on AKI remain a research focus in nephrology, investigations into the susceptibility mechanisms and therapeutic targets of AKI in the elderly remain limited. Therefore, elucidating the underlying mechanisms driving the higher incidence and severity of AKI in elderly patients is crucial. Furthermore, identifying more sensitive biomarkers and targeted therapeutic strategies is essential for improving outcomes in this population.

AKI has diverse etiologies. Cisplatin, a commonly used chemotherapeutic agent, induces nephrotoxicity, which represents one of its most frequent adverse effects. The incidence of AKI in elderly patients receiving cisplatin is 2.96 times higher than that in younger patients [1]. To investigate this age-related susceptibility, we utilized a cisplatin-induced AKI model. We observed that aged mice (O-CIS group) developed AKI with significant renal function decline following low-dose cisplatin administration, whereas young mice (Y-CIS group) showed no significant injury (Fig. 1A and 1B). The renal injury in aged mice was more serious than that in young mice (Fig. 1C–F). We also measured the body weight of both young and old mice. The results showed no significant difference in body weight between the different age groups of AKI mouse models, thus ruling out the potential influence of the total dose of cisplatin on the experimental outcomes (Fig. S1). The mechanisms contributing to the susceptibility to cisplatin-induced AKI in the elderly are complex, with enhanced oxidative stress being a key focus in both the aging process and AKI pathogenesis. Mitochondrial metabolism is closely linked to oxidative stress, as reactive oxygen species (ROS) generated during mitochondrial metabolism can, when overproduced, exacerbate oxidative stress, which can drive or accelerate aging in physiological senescence or age-related diseases. Comparative analyses of oxidative stress levels further demonstrate significantly elevated renal ROS (Fig. 1G and 1H) and decreased reduced glutathione (GSH) (Fig. S2A) in aged cisplatin-induced AKI mice.

**Figure 1.**
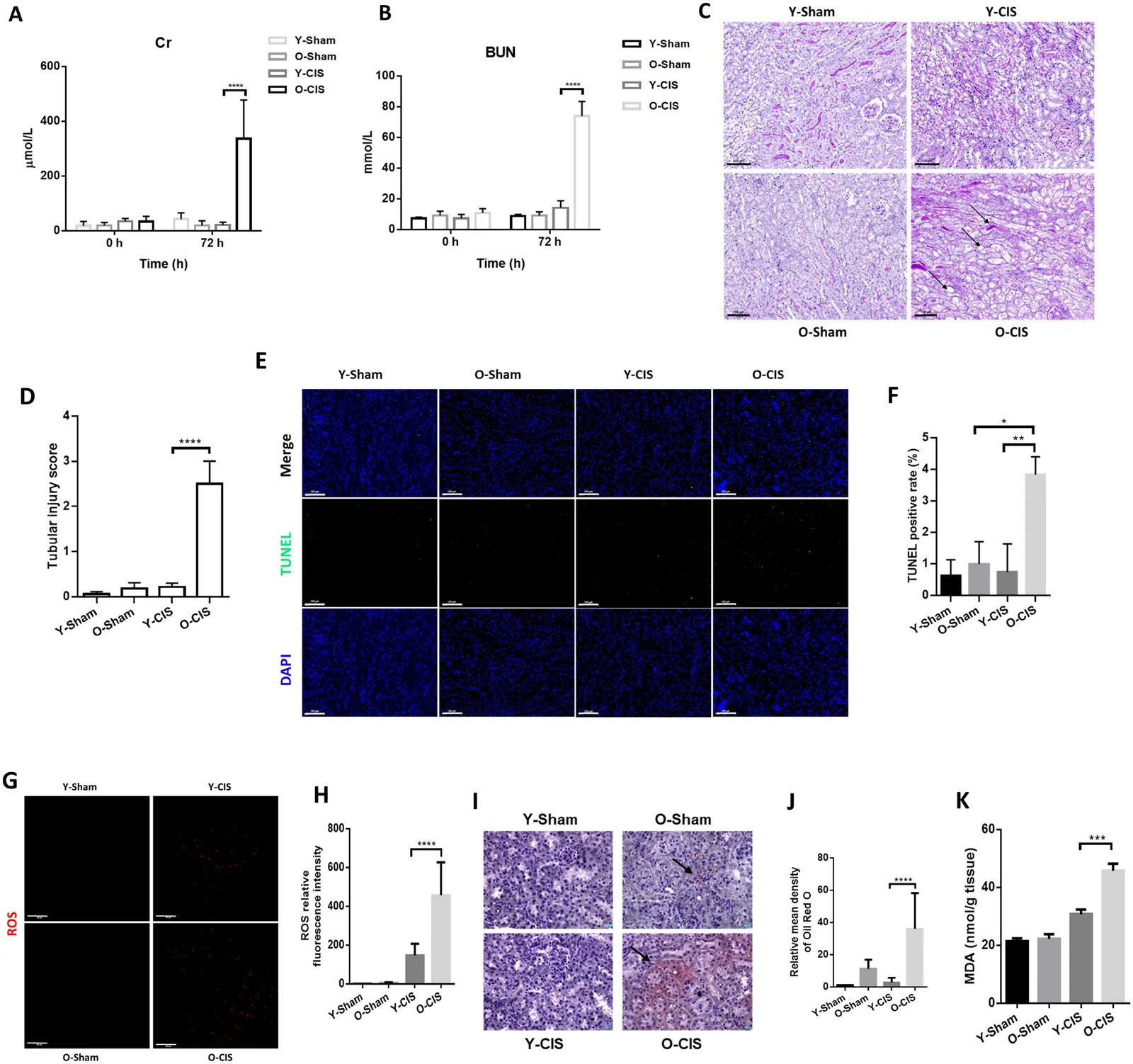
The kidneys of aged mice exhibit greater susceptibility to AKI with more severe oxidative stress and lipid metabolic disorders. (A) Serum creatinine. (B) Blood urea nitrogen. (C) Representative images of renal PAS staining. Magnification 200×. The black arrows indicate brush border abscission of the renal tubules, tube cast, degeneration and necrosis of the renal tubules. Scale bar = 100 μm. (D) Histology score. (E and F) Representative images of TUNEL staining. Magnification 400×. Scale bar = 100 μm. (G and H) The level of ROS was assessed by DHE staining. Magnification 400×. Scale bar = 50 μm. (I and J) Oil red O staining was used to assess lipid deposition. Scale bar = 50 μm. (K) MDA in tissue. The values are the means ± SEM. *n* = 5 animals per group. *P < 0.05; **P < 0.01; ***P < 0.001; ****P < 0.0001. Y, young; O, old; CIS, cisplatin. Abbreviations: DHE, dihydroethidium; MDA, malondialdehyde; PAS, periodic acid-Schiff staining; ROS, reactive oxygen species; TUNEL, terminal deoxynucleotidyl transferase-mediated dUTP nick-end labeling. Bar graphs and representative tissue staining microgra- phs across panels A to K show greater cisplatin-induced acute kidney injury, oxidative stress and lipid metabolic disorders in aged mice.

Renal cells, especially renal tubular epithelial cells (RTECs), primarily rely on mitochondrial fatty acid β-oxidation for energy production. Following AKI, impaired fatty acid oxidation leads to the accumulation of lipotoxic metabolites such as ceramide and malondialdehyde (MDA), which subsequently enhance ROS production and promote mitochondrial dysfunction. Notably, mitochondrial dysfunction serves as a major source of ROS, which induces the release of lipid radicals from triglycerides or phosphatidylcholine, further aggravating lipotoxicity, oxidative stress, and mitochondrial damage. Ultimately, this cascade promotes cellular death and establishes a vicious cycle. A similar suppression of fatty acid oxidation and concomitant lipid accumulation has been observed in cisplatin-induced AKI models [2]. The aging process is also associated with lipid metabolic dysregulation, which accelerates senescence and the progression of age-related diseases. In aged renal cells, key proteins involved in fatty acid metabolic pathways are downregulated, and mitochondrial ROS production becomes more pronounced [3]. These findings suggest that lipid metabolic dysregulation may exacerbate oxidative stress, thereby functioning as a critical factor underlying the increased susceptibility to cisplatin-induced AKI in the elderly. To investigate this hypothesis, we performed Oil Red O staining and measured MDA and 4-hydroxynonenal (4-HNE) levels in the kidneys of young and aged mice with cisplatin-induced AKI (Figs. 1I–K and S2B–D). The results revealed markedly elevated renal lipid accumulation and lipid peroxidation in RTECs of aged mice following equivalent cisplatin exposure. We also performed PCR analysis on several genes involved in lipid metabolism (Fig. S2E). The results revealed substantial differences in the expression of multiple lipid metabolism-related genes, such as peroxisome proliferator-activated receptor α (*Ppar*α) and carnitine palmitoyltransferase 1α (*Cpt1*α) between young and aged AKI models. These findings further suggest that dysregulated lipid metabolism may contribute to the increased susceptibility to AKI in the aged kidney (Fig. S2F).

Excessive accumulation of free fatty acids (FFAs) or their metabolic intermediates induces severe cytotoxicity and cellular damage. Consequently, most cells typically store fatty acids in LDs. As organelles, LDs sequester FFAs into a confined space, effectively mitigating lipid peroxidation and lipotoxicity. Therefore, LDs can exert a protective effect on the cell to some extent. However, persistent excessive LD accumulation reflects disrupted cellular homeostasis and potentially progresses to cellular steatosis. Accumulating evidence demonstrates LD accumulation in senescent cells. Immunohistochemical analysis of the LD marker Perilipin 2 (PLIN2), we detected significantly PLIN2 accumulation in aged mouse kidneys after injury (Fig. 2A and 2B). Therefore, LDs homeostasis represents a critical process for maintaining cellular lipid balance and may play a regulatory role in AKI in the elderly. Evidence suggests that LDs facilitate FFA transfer to mitochondria through LD–mitochondria contacts. This mechanism enhances lipid trafficking efficiency while minimizing cytoplasmic FFA exposure, consequently reducing toxic lipid accumulation, exerting cytoprotective effects, and promoting fatty acid β-oxidation [4]. Hence, modulating LD–mitochondria contacts is crucial for sustaining lipid homeostasis, optimizing mitochondrial β-oxidation, and attenuating oxidative stress. However, LD–mitochondria contacts remain unexplored in aging systems. To evaluate LD–mitochondria contacts, immunofluorescence co-staining of PLIN2 (green) and the mitochondrial marker translocase of outer mitochondrial membrane 20 (Tomm20) (red) was performed. Quantitative analysis revealed significantly reduced LD–mitochondria contacts (yellow) in aged cisplatin-induced AKI mouse kidneys (Fig. 2C and 2D).

**Figure 2.**
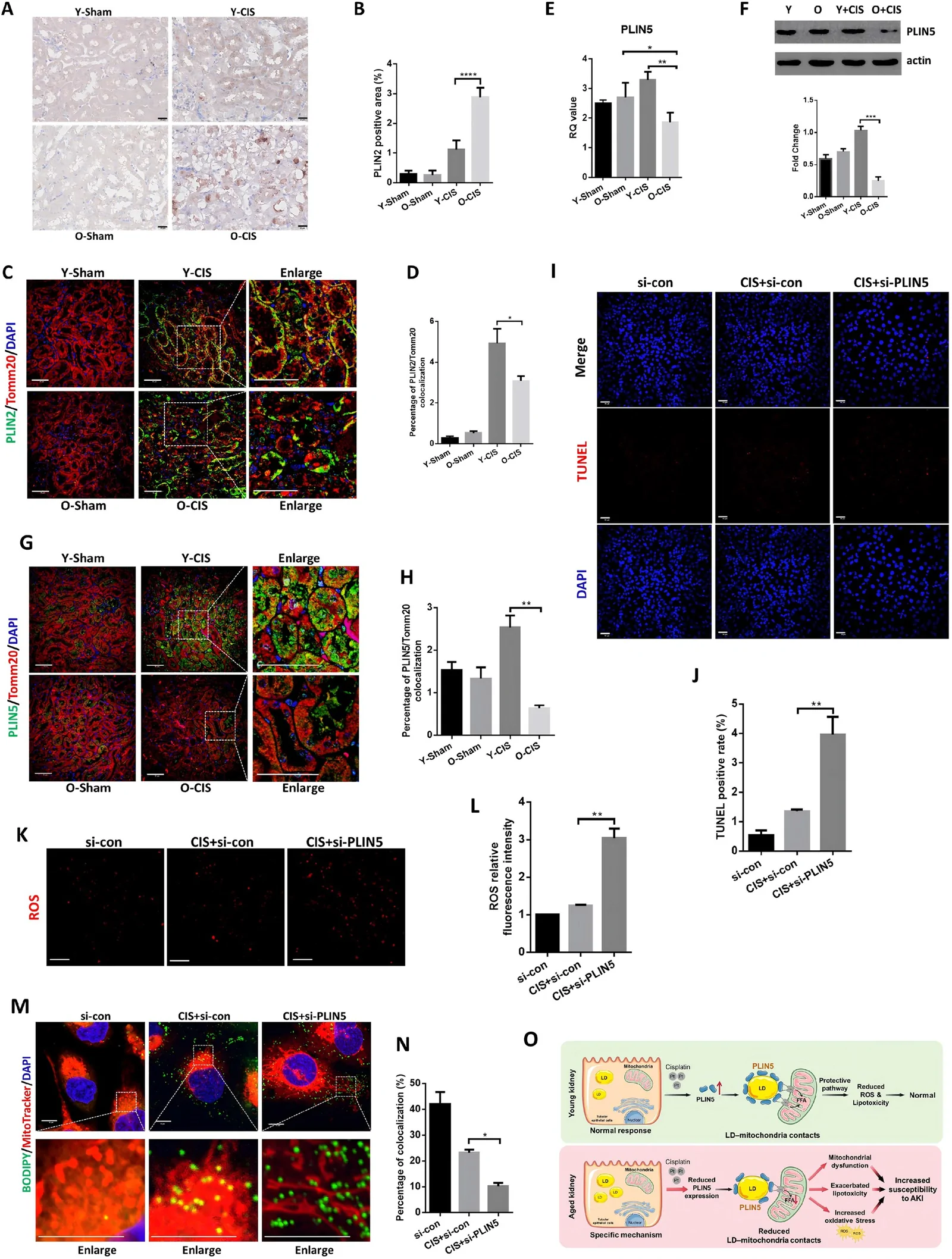
PLIN5-mediated LD–mitochondria contacts influenced the susceptibility of RTECs to cisplatin-induced injury. (A and B) IHC staining analysis of PLIN2 in tissue. Magnification 200×. Scale bar = 20 μm. (C and D) Immunofluorescence co-staining of PLIN2 (green) and mitochondrial marker Tomm20 (red) analysis of the integrity of the LD–mitochondria contacts. Magnification 400×. Scale bar = 50 μm. (E and F) PLIN5 expression in the kidney. (G and H) Immunofluorescence co-staining analysis of PLIN5 and mitochondrial marker Tomm20. Magnification 400×. Scale bar = 50 μm. (I and J) TUNEL staining in human renal tubular epithelial cell line (HRTECs). Magnification 400×. Scale bar = 50 μm. (K and L) The level of ROS was assessed by DHE staining in HRTECs. Magnification 400×. Scale bar = 50 μm. (M and N) Immunofluorescence co-staining for MitoTracker and BODIPY to assess the integrity of the LD–mitochondria contacts in HRTECs. Scale bar = 10 μm. (O) Graphic summary. The values are the means ± SEM. *n* = 5 animals per group. *P < 0.05; **P < 0.01; ***P < 0.001; ****P < 0.0001. Y, young; O, old; CIS, cisplatin. Abbreviations: PLIN2, Perilipin 2; PLIN5, Perilipin 5; Tomm20, translocase of outer mitochondrial membrane 20; TUNEL, terminal deoxynucleotidyl transferase-mediated dUTP nick-end labeling. Immunohistochemistry, immunofluorescence, bar graphs and a graphic summary across panels A to O demonstrate that PLIN5-mediated lipid droplet-mitochondria contacts modulate cisplatin-induced injury susceptibility in renal tubular epithelial cells.

Our results indicate that LDs are slightly increased in the kidneys of aged mice under basal conditions. Moderate LD formation may mitigate lipotoxicity and no excessive LDs accumulation was observed at this stage. Furthermore, no significant differences were observed in PLIN2 expression, levels of lipid peroxidation products (MDA and 4-HNE), or overall oxidative stress status between Y-Sham and O-Sham groups. These results suggest that lipid metabolism is maintained in a dynamic equilibrium in both young and aged kidneys under physiological conditions. Immunofluorescence co-staining for PLIN2 (green) and the mitochondrial marker Tomm20 (red) revealed low expression levels in the kidneys of both young and aged mice under physiological conditions, further suggesting that lipid metabolism at this stage may not require excessive mobilization of LD–mitochondria contacts. However, following cisplatin stimulation, these parameters changed significantly in aged mice compared with those in young mice, indicating that LD–mitochondria contact-mediated dysregulation of lipid metabolism is an important process in the susceptibility to AKI with aging.

Perilipin 5 (PLIN5), enriched on the surface of LDs adjacent to mitochondria, induces mitochondrial recruitment to LD peripheries and is a critical protein mediating LD–mitochondria contacts [5]. As a member of the Perilipin family, PLIN5 primarily participates in LD formation, stabilization, and metabolic regulation. By modulating interactions among LDs surface proteins, PLIN5 influences lipid breakdown and storage, thereby preventing uncontrolled lipolysis. For example, PLIN5 can suppress adipose triglyceride lipase activity, reducing lipolysis and regulating triglyceride storage and release within LDs. This mechanism maintains fatty acid metabolic demand while preventing the accumulation of harmful lipid peroxides. PLIN5 overexpression can also enhance fatty acid oxidation rates and improve LD–mitochondria contacts integrity [6]. These functions are important for mitigating mitochondrial damage caused by FFA peroxidation and alleviating oxidative stress. Direct evidence addressing PLIN5 functionality in aging is indeed limited. Existing studies primarily document tissue-specific changes in PLIN5 expression levels with age. For instance, PLIN5 expression was downregulated in cardiomyocytes isolated from aged mice, while it showed no significant change in muscle tissue from healthy humans of different ages [7] or in the aging brain [8]. Notably, a study in murine brown adipose tissue observed a significant age-related reduction in the average volume and surface area of individual LD–mitochondria contact sites [9]. However, the role of LD–mitochondria contacts in kidney disease remains poorly understood, and no studies have investigated PLIN5-mediated LD–mitochondria contacts in cisplatin-induced AKI, particularly regarding age-related susceptibility. Therefore, we examined PLIN5 expression at the transcriptional and protein levels. Results revealed no significant difference in renal PLIN5 expression between young and aged mice under basal conditions. However, cisplatin stimulation markedly increased PLIN5 levels in young mice but significantly decreased them in aged mice (Fig. 2E and 2F). This result was interesting. Immunofluorescence co-staining demonstrated reduced PLIN5-mitochondria colocalization in aged mice with cisplatin-induced AKI (Fig. 2G and 2H). PLIN5 knockdown in human renal tubular epithelial cells (HRTECs) exacerbated cisplatin-induced cellular injury (Fig. 2I and 2J) and ROS (Fig. 2K and 2L) and diminished LD–mitochondria contacts (Fig. 2M and 2N). To further validate the protective role of PLIN5 against age-related susceptibility to AKI, we overexpressed PLIN5 via adeno-associated virus (AAV) in aged mice, induced an AKI model, and examined tissue injury markers. The results demonstrated that upregulation of PLIN5 mitigated cisplatin-induced declines in renal function, renal pathological damage, oxidative stress, and LD accumulation (Fig. S3A–F and S3H–K). The co-localization of PLIN5 with mitochondria was increased, suggesting enhanced LD–mitochondria contacts (Fig. S3G and S3L). This finding further indicates the critical role of PLIN5-mediated LD–mitochondria contacts in cisplatin-induced AKI susceptibility.

However, the underlying mechanism driving the reduction of PLIN5 in aged kidneys following injury remains unclear. Previous research indicates that *Ppar*α expression is significantly decreased and fatty acid oxidation is impaired in the kidneys of aged mice. Given that *Ppar*α is a transcription factor of PLIN5, we assessed *Ppar*α expression to investigate whether it contributes to the downregulation of PLIN5 in the aged kidney. While no significant decrease was observed in aged kidneys in the Sham group, its expression was markedly lower in aged mice compared to young mice following cisplatin injection (Fig. S2E). This finding suggests a potential role for *Ppar*α in the injury-specific downregulation of PLIN5. Although altered *Ppar*α expression may partially explain the decrease in PLIN5, the potential involvement of post-transcriptional or post-translational modifications in regulating PLIN5 in AKI cannot be excluded. Meanwhile, age-associated mitochondrial dysfunction may also contribute to impaired fatty acid oxidation. To better define the functional role of PLIN5, more direct assessments of mitochondrial function such as Seahorse, as well as fatty acid oxidation flux are warranted. Elucidating these precise mechanisms will be a central focus of our future investigations.

These results indicated that young and aged kidneys exhibited differential responses in lipid metabolic regulation following the same stimulation. Following cisplatin-induced injury, aged kidneys demonstrated a specific downregulation of PLIN5, which triggered abnormal LD–mitochondria contacts. This exacerbated lipotoxicity, mitochondrial dysfunction, and oxidative stress, ultimately rendering aged kidneys more susceptible to cisplatin-induced AKI (Fig. 2O). Our findings provide direction for subsequent research. Elucidating the intrinsic mechanism of PLIN5-specific reduction and modulating PLIN5-mediated LD–mitochondria contacts may serve as a therapeutic approach to mitigate cisplatin-induced AKI susceptibility in the elderly.

## Research limitations

A limitation of this study is that only aged male mice were utilized. It is well-established that significant sex differences exist in aging and susceptibility to AKI. Furthermore, the common etiologies of AKI and the influence of various hormones on AKI susceptibility can differ substantially between sexes [10]. Consequently, our findings are primarily applicable to the male context, and their generalizability requires future validation in female populations. Elucidating these sex-dependent mechanisms will provide a crucial additional dimension to our understanding of age-related kidney disease. On the other hand, it is important to note that this study primarily relied on a cisplatin-induced model of AKI. While this model is well-established for studying drug-induced tubular injury, it may not fully recapitulate the complexity of AKI in the elderly, which often arises from multifactorial causes such as sepsis or renal hypoperfusion [1, 2]. Therefore, the therapeutic potential of PLIN5 modulation, requires further investigation in additional preclinical models. Determining whether the protective role of PLIN5 extends to these contexts will be crucial for assessing its broad applicability as a therapeutic target.

## Supplementary Material

lnag025_Supplementary_Data
