# Supplementary material for: PLIN5-mediated lipid droplet–mitochondria contacts dysregulation underlies the increased susceptibility to acute kidney injury with aging
Source: Life Med. 2026 Jul 8;5(4):lnag025. doi: 10.1093/lifemedi/lnag025 (PMC13521333; doi:10.1093/lifemedi/lnag025)
Supplement: Supplementary file 1 [file lnag025_supplementary_data.docx]

**Title:** **PLIN5-mediated lipid droplet-mitochondria contacts dysregulation underlies the increased susceptibility to acute kidney injury with aging**

Siyang Wang^1,2,*^. Jie Gao^3^. Zhuo Li^3^. Yue Xu^1^. Guangyan Cai^1,*^. Xiangmei Chen^1,*^

^1^Department of Nephrology, the First Medical Centre, Chinese PLA General Hospital, Chinese PLA Institute of Nephrology, State Key Laboratory of Kidney Diseases, National Clinical Research Center for Kidney Diseases, Beijing 100853, China

^2^Department of Emergency Medicine, Xinqiao Hospital, Army Medical University (Third Military Medical University), Chongqing 400037, China

^3^Department of Nephrology, Shandong Provincial Hospital Affiliated to Shandong First Medical University, Jinan 250021, China

***Correspondence:** xmchen301@126.com (X.C.), caiguangyan@sina.com (G.C.), 378478324@qq.com (S.W.)

**Supplemental figures and figure legends**

**Figure S1. Body weight of mice.**

Body weight of mice in different groups. The values are the means ± SEM. Y, young; O, old; CIS, cisplatin.

**Figure S2. Redox status and lipid metabolism disorders in the kidney tissues of mice from different groups.**

(A) Reduced Glutathione (GSH) in tissue. (B–D) 4-hydroxynonenal (4-HNE) in tissue. Scale bar = 20 μm. (E) Real-time PCR analysis of peroxisome proliferators-activated receptor α (PPARα)，peroxisome proliferators-activated receptor γ coactivator lα (PGC-1α), carnitine palmitoyltransferase 1α (CPT1α), Fatty Acid Transport Protein 2 (FATP2) and CD36 in renal tissue. (F) Western blot analysis of PPARα expression in renal tissue. The values are the means ± SEM. **P* < 0.05; ***P* < 0.01; ****P* < 0.001; *****P* < 0.0001. Y, young; O, old; CIS, cisplatin.

**Figure S3. AAV-mediated overexpression of PLIN5 protects against renal damage in cisplatin-induced AKI in aged mice**

(A) Blood urea nitrogen. (B) Serum creatinine. (C, H) Representative images of renal PAS staining and histology score. Magnification 200×. Scale bar = 50 μm. (D, I) Representative images of TUNEL staining. Scale bar = 100 μm. (E, J) The level of ROS was assessed by DHE staining. Scale bar = 100 μm. (F, K) Oil red O staining was used to assess lipid deposition. Scale bar = 50 μm. (G, L) Immunofluorescence costaining analysis of PLIN5 (green) and mitochondria marker Tomm20 (red). Magnification 400×. Scale bar = 50 μm. The values are the means ± SEM. *n* = 5 animals per group. **P* < 0.05; ***P* < 0.01; *** *P* < 0.001; O, old; CIS, cisplatin; AAV, adeno-associated virus. Abbreviations: PAS, periodic acid-schiff staining; TUNEL, TdT mediated dUTP Nick End Labeling; ROS, reactive oxygen species; DHE, Dihydroethidium; PLIN5, Perilipin 5; Tomm20, translocase of outer mitochondrial membrane 20.

**SUPPLEMENTAL DATA**

**Table S1. Primer sequences**.

| Gene | Species | Forward primer | Reverse primer |
| --- | --- | --- | --- |
| *PLIN5* | Mouse | CAGAGCAAACACCGTACCCAG | GGGATGGAAAGTAGGGCTAGG |
| *PLIN5* | Human | TGCCCATGACTGAAGCTGAG | ACGCACAAAGTAGCCCTGTT |
| *PPARα* | Mouse | GGGCAAGAGAATCCACGAAG | GTTGTTGCTGGTCTTTCCCG |
| *PGC-1α* | Mouse | GACAGCTTTCTGGGTGGATT | CGCAGGCTCATTGTTGTACT |
| *CPT1α* | Mouse | GGTCTTCTCGGGTCGAAAGC | TCCTCCCACCAGTCACTCAC |
| *FATP2* | Mouse | ATGCTGGAGGAAAGACCCAGAC | CACGCCATACACATTCACTTCTTC |
| *CD36* | Mouse | GCTTATTGGGAAGACAATCAA | GCAAATGTCAGAGGAAAAGAA |

**Supplemental Methods**

**Ethics approval**

The animal care and experimental procedures were approved by the ethics committees for animal experimentation of Army Medical University (No. AMUWEC20224372) and conducted in accordance with the Guide for the Care and Use of Laboratory Animals published by the US National Institutes of Health (NIH Publication No. 85-23, revised 2011).

**Animals**

All animal procedures carried out in this study were approved by the Animal Care and Use Committee of the Chinese PLA General Hospital and conducted in accordance with the Guide for the Care and Use of Laboratory Animals published by the US National Institutes of Health (NIH Publication No. 85-23, revised 2011). Three-month-old and 25-month-old male C57BL/6 mice were purchased and housed at the Experimental Animal Centre of the Chinese PLA General Hospital. Then, the mice were randomized into the young control (Y), old control (O), young mice with cisplatin injection (Y-CIS), old mice with cisplatin injection (O-CIS). All animals were given free access to food and water and were maintained under a 12-h light/dark cycle throughout the study. To study the effects of cisplatin on the kidney, we intraperitoneally injected aged mice with cisplatin (11 mg/kg). The control group was injected with normal saline.

To induce PLIN5 overexpression in mice, we utilized adeno-associated virus (AAV) vectors to introduce PLIN5 into the kidney via tail vein injection. 24-month-old male C57BL/6 mice were divided into 3 groups: control (Sham), cisplatin injection (CIS) and PLIN5 overexpression in mice with cisplatin injection (AAV-PLIN5+CIS). Sham and CIS groups were injected with AAV-control via the tail vein, AAV-PLIN5+CIS group was injected with AAV-PLIN5 (1×10^12^ viral genomes·mL−1). After 3 weeks, the AKI model was established in CIS and AAV-PLIN5+CIS groups of mice.

**Cell culture**

Human renal tubular epithelial cells (HRTECs) were purchased from ATCC. Cells were cultured cultured in F12/DMEM with 10% FBS (Gibco) in a humidified atmosphere of CO_2_/ air (5%/95%) at 37°C. siRNA PLIN5 and control (GenePharma, Shanghai, China) were transfected into cells according to the manufacturer’s protocol. Twenty-four hours after transfection, cisplatin (2 mg/mL) was added to the medium to stimulate cells for 24 h. cells from different groups were costained with MitoTracker and BODIPY for 30 min and then photographed under a fluorescence microscope. BODIPY is used to specifically stain lipid droplets composed of neutral lipids, thereby enabling their visualization in cellular studies.

**Serum biochemistry analysis**

Serum samples were collected by centrifugation at 3000 rpm for 10 min and stored at −80°C before analyses. Serum biochemical parameters were measured using an autoanalyser (Cobas 8000, Roche, Germany).

**Renal histopathological studies**

Kidneys were excised, fixed in 4% paraformaldehyde and embedded in paraffin. For light microscopy, paraffin embedded renal sections (4 mm thick) were stained with periodic acid-Schiff (PAS) solution and examined under a microscope.

**Western blot analysis**

Frozen kidney tissues were lysed in RIPA lysis buffer and centrifuged at 12,000 × *g* at 4℃ for 30 min to obtain cellular proteins in the supernatant. Equal amounts of protein from each sample were resolved by sodium dodecyl sulfonate–polyacrylamide gel electrophoresis (SDS-PAGE) and transferred to nitrocellulose membranes, which were blocked with 5% skim milk for 1 h at room temperature. The membranes were then probed with primary antibodies at 4℃ overnight and subsequently probed with horseradish peroxidase-conjugated anti-rabbit IgG (Beyotime Biotechnology) at dilutions of 1:1000–1:5000. Immunoreactive bands were visualized by enhanced chemiluminescence, and densitometry was performed using Quantity One software (Bio-Rad Laboratories).

**TUNEL**

TUNEL staining was performed with a cell death detection kit (Roche) according to the manufacturer’s instructions. TUNEL-positive tubular cells were counted in ten non-overlapping fields of each sample.

**RNA isolation and real‑time PCR**

Total RNA was isolated from renal tissues using TRIzol (Invitrogen, Carlsbad, CA) according to the manufacturer’s instructions. A UV spectrophotometer was used to measure the concentrations of total RNA. To measure mRNA, 1 μg RNA was reversely transcribed using a cDNA Reverse Transcription Kit (Takara), and real-time PCR was performed using SYBR Green PCR Master Mix (Takara) according to the manufacturers protocol. The primer sequences are in Table S1. 18S was used for normalization. Relative quantification was performed using the ΔCt method. PCR analyses were conducted using an Applied Biosystems ABI Prism 7500 Sequence Detection.

**Immunohistochemistry**

Immunohistochemistry was performed with IHCeasy PLIN2 Ready-To-Use IHC Kit (Proteintech) and IHC Prep Detect Kit (Proteintech). The results were quantified by Image J software, and the average value of each group was used to determine the ratio relative to the control group.

**Immunofluorescence**

Briefly, after deparaffinization, hydration, antigen repair, and blocking, the tissue sections were incubated with different antibodies overnight at 4°C, followed by incubation with Cy3- or FITC-conjugated secondary antibodies (Beyotime) at 37°C for 1 h. The nuclei were counterstained with DAPI. They were viewed and captured by confocal microscope Olympus Fluoview FV3000. The immunofluorescence intensity and fluorescence colocalization of the target proteins were calculated with ImageJ software.

**Oil Red O Staining.**

Renal frozen sections incubated in the dark with oil red dye for 10 min, and subsequentlywashed with 60% isopropanol. After nuclear staining and sealing, the slides were observed and photographed under a microscope and analyzed with Image J software.

**ROS Staining.**

ROS levels were determined by ROS Kit (Bestbio) according to the manufacturer’s instructions. In brief, the tissue sections were incubated with DHE at room temperature for 1 h, photographed under confocal microscope Olympus Fluoview FV3000, and analyzed with Image J software.

**Statistical analysis**

All data were analysed using SPSS 20.0 (SPSS, Chicago, IL). Comparisons among groups were analysed by analysis of variance (ANOVA), and values of *P* < 0.05 indicated statistical significance.

**Data availability**

The datasets used and/or analysed during the current study are available from the corresponding author on reasonable request.
